# Image-Guided Thermal Ablation of Stage 1 Single and Multiple Primary Lung Carcinoma: Five-Year Outcomes

**DOI:** 10.3390/medsci14020272

**Published:** 2026-05-27

**Authors:** Jamie E. Clarke, Noor Jahanshahi, Bianca Villegas, Grace Hyun J. Kim, Soheil Kooraki, Matthew Quirk, Scott Genshaft, Robert D. Suh, Fereidoun Abtin

**Affiliations:** 1 Department of Radiological Sciences, University of California Los Angeles, 757 Westwood Plaza, Suite 1621, Los Angeles, CA 90095, USAfabtin@mednet.ucla.edu (F.A.); 2 School of Medicine, Creighton University, 2500 California Plaza, Omaha, NE 68178, USA; 3 Computer Vision and Imaging Biomarkers Laboratory, University of California, Los Angeles, CA 90095, USA

**Keywords:** cancer, lung ablation, CT-guided, radiology, oncology, pulmonary

## Abstract

Background: Image-guided thermal ablation has been used for the treatment of primary lung carcinoma but its use in the treatment of multiple lung carcinoma and effects on survival have not been well established. Objective: This study compares the long-term survival metrics for stage 1 single primary lung cancer and multiple primary lung cancer (MPLC) in patients treated with image-guided thermal ablation (IGTA). Methods: A retrospective institutional review included 37 NSCLC patients (mean age 71.6 ± 8.8 years) with ≥5 years follow-up. In total, 119 IGTA procedures were performed. Among patients with a single tumor (n = 14, 37.8%), each underwent a single ablation session. In contrast, patients with MPLC (n = 23, 62.2%) underwent 88 ablation sessions to treat 105 tumors. Data included demographics, tumor features, procedural details, safety, adverse events, and outcomes. Primary endpoints were 5-year overall survival (OS), progression-free survival (PFS), and cancer-specific survival (CSS). Results: All ablations were completed successfully. Severe AEs occurred in 5.8% (7/119) of the ablations and were limited to pneumothorax requiring chest tube placement with hospitalization. At the time of ablation, individual nodules were staged at T1A = 46 (38.7%), T1B = 54 (45.4%), T1C = 16 (13.5%) and T2A = 3 (2.5%). Local recurrence was observed in 4/119 (3.3%) ablated tumors, all at stage T1B, and all were retreated with ablation. The 5-year OS was better for patients with MPLC at 85.6% compared to patients with a single tumor at 35.7% (HR = 0.14, *p* = 0.003, 95% CI: 0.037, 0.51). The 5-year OS for tumors based on T classification for T1A, T1B, TIC and T2A was 71.4%, 66.8%,66.7% and 0%. The 5-year PFS was 77.4% for patients with MPLC compared to 35.7% for patients with single primary lung cancer (HR = 0.25, *p* = 0.014, 95% CI: 0.084, 0.76). The 5-year CSS was 95.2% for patients with MPLC compared to 83.1% for patients with single primary lung cancer (HR = 0.21, *p* = 0.16, 95% CI: 0.018, 2.33). Conclusions: IGTA is an effective and safe treatment for patients with stage 1 single primary lung cancer and MPLC with limited local recurrence. Tumor size up to 3 cm did not have significant impact on survival. Overall survival was improved in patients with MPLC compared to those with single NSCLC. Clinical Impact: IGTA can be safely performed in patients with single primary lung cancer and MPLC, with limited local recurrence rate. Highlights: Key Findings: IGTA effectively treats patients with stage 1 single primary lung cancer and MPLC, with 3.3% recurrence, which can be retreated with ablation. The five-year OS was higher in patients with MPLC (85.6%) versus those with single lung cancer (35.7%, *p* = 0.003). OS by T classification: 71.4% for T1A, 66.8% for T1B, 66.7% for TIC, and 0% for T2A. Importance: IGTA effectively treats patients with single primary lung cancer and MPLC with low recurrence. Tumor size < 3 cm showed no impact on overall survival.

## 1. Introduction

Lung cancer still remains the leading cause of cancer-related mortality in the United States, claiming approximately 131 thousand lives annually [1]. Surgical resection has been the mainstay of treatment for early-stage non-small cell lung cancer (NSCLC) [2]. In the past few decades, non-surgical treatment options such as stereotactic ablative radiotherapy (SABR) and image-guided thermal ablation (IGTA) have become popular alternatives to surgery.

IGTA involves heating tissue, using radiofrequency ablation (RFA) or microwave ablation (MWA), or freezing tissue through cryoablation (CA) [3]. The use of thermal ablation for lung neoplasms has been repeatedly described as safe, feasible, and efficient in an outpatient setting; however, previous studies have primarily focused on technical aspects of the ablation and survival outcomes in single lung carcinoma with limited investigation into treatment and survival outcomes in MPLC [4,5,6,7,8,9,10,11].

Following the National Lung Screening Trial (NLST) and the adoption of lung cancer screening (LCS) by the United States Preventive Service Task Force (USPSTF) and other agencies, there has been an ever-increasing detection of lung cancer in the screened population [12,13]. Among the cancers detected in the LCS cohort, stage 1 lung cancer constitutes up to 50% of the screened population [14]. Surgical resection remains the therapy of choice for lung cancer, although not all lung cancers are amenable to resection. Based on the National Comprehensive Cancer Network (NCCN) guidelines, image-guided thermal ablation is a viable alternative in select patients with stage 1 NSCLC who are deemed medically inoperable due to underlying comorbidities or insufficient pulmonary functional reserve and negative mediastinal nodal disease [15].

There is increasing recognition of MPLC detected at the time of screening or following management and follow-up of treated lung carcinoma which can range from 3.72% to 12% [16]. These patients with MPLC include second primary lung carcinoma, intraparenchymal metastasis, multifocal lung adenocarcinoma with ground-glass or lepidic patterns and pneumonic-type lung cancer and are defined as synchronous or metachronous lung cancers [16]. In the metachronous group, a time-of-presentation interval of 2 years is required between tumors [17]. Patients with second primary lung carcinomas and multifocal lung adenocarcinomas are often amenable to image-guided thermal ablation (IGTA), which is recognized and incorporated within the NCCN guidelines [15].

The purpose of the present study is to assess the long-term outcomes of patients with stage 1 non-small cell lung cancer who underwent curative-intent IGTA for single tumors or MPLC using IGTA. The study aims to evaluate the overall 5-year survival (OS), progression-free survival (PFS), and the cancer-specific survival (CSS) rates following IGTA.

## 2. Materials and Methods

### 2.1. Patient Selection

The study was conducted according to the guidelines of the Declaration of Helsinki, and approved by the Institutional Review Board of the University of California, Los Angeles (UCLA) (protocol code 12-1270 and date of approval: 22 August 2012). This Institutional Review Board (IRB)-approved, Health Insurance Portability and Accountability Act (HIPAA)-compliant, single-center, retrospective study included patients who received IGTA and had 5-year follow-up at a tertiary referral center in the United States. Inclusion criteria consisted of patients who (i) had stage 1 NSCLC confirmed through biopsy, (ii) were deemed ineligible for surgical resection of the primary tumor or refused surgery, (iii) were above 18 years of age, and (iv) were not previously locally treated for the target tumor (i.e., recurrence after radiation, surgery or IGTA). Ineligibility for surgery was primarily due to insufficient pulmonary reserve, which resulted from prior lung surgery or radiotherapy for a separate lung malignancy or other comorbidities, including COPD or heart disease, which further prevented the patient from tolerating surgery. Comorbidity was evaluated according to the Adult Comorbidity Evaluation-27 (ACE-27) scoring system at the time of initial presentation for consideration of a lung ablation procedure as a standard element of documentation in the medical records at our institution. Early-stage NSCLC was defined as stage IA tumors of ≤ 3 cm in diameter and stage IB tumors of 3–5 cm, confined to the lung with no clinical or imaging evidence of nodal or distant metastasis (T1 and T2A, N0, M0).

**Figure 1:**
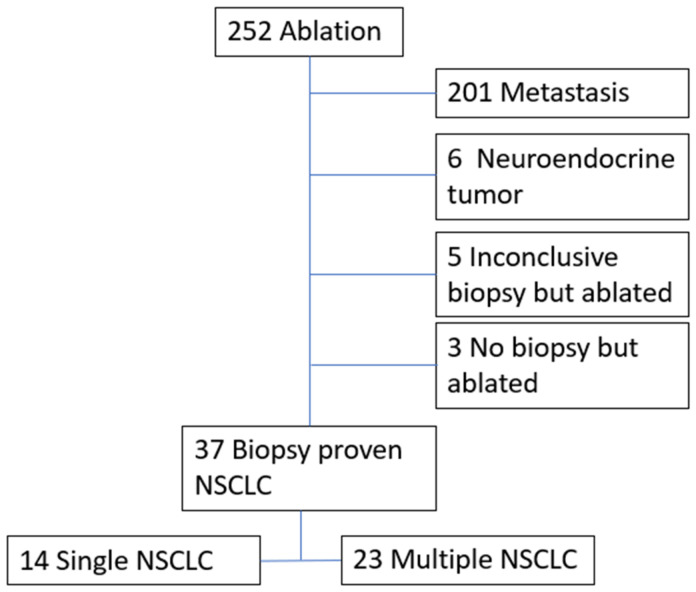
Flow chart of ablations and patient selection.

From 252 ablations, 37 patients (21 females, 16 males) with mean age of 71 years (ranged 44–88) qualified for analysis (Figure 1). Patients were identified through the weekly institutional multidisciplinary thoracic oncology tumor board, where experts in interventional radiology, thoracic surgery, medical oncology, radiation oncology, and pathology collectively discussed the cases. The decision to proceed with thermal ablation was made through joint opinions and discussions involving the patient. For patients with MPLC, only a single consultation and biopsy was performed on the initial targeted tumor to confirm NSCLC. Thereafter, the tumors were not biopsied and directly underwent ablation based on changes in imaging characteristics which included; growth beyond 20% compared to baseline CT scans, increased density of the nodule or part of the nodule from ground-glass to part-solid or solid, increased thickness of the wall of cystic nodules or increased multilocation and size of cystic nodules, or increase 18F-Fluorodeoxyglucose (FDG) uptake and metabolic activity. Confirmatory computed tomography (CT) scans were performed on the day of the procedure for each patient. Demographic data, prior history of SABR, surgery, and chemotherapy for prior primary NSCLC or other cancers and cause of death were recorded.

### 2.2. Ablation Techniques

**Figure 2:**
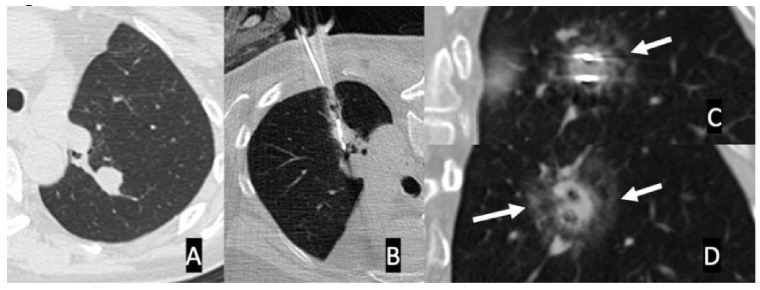
(**A**) Axial CT scan image demonstrating a 23 × 12 mm left lower lobe biopsy-proven adenocarcinoma. (**B**) Axial images with the patient prone show two antennas placed parallel with the tips extending approximately 5 mm beyond the tumor. (**C**) Coronal image following MWA using 30 W for 5 min shows a faint halo of ground-glass surrounding the tumor and confirming the ablation zone (arrow). (**D**) Following removal of the antennas, the ground-glass is better visualized and confirms adequate coverage following MWA (arrows) surrounding the tumor.

**Figure 3:**
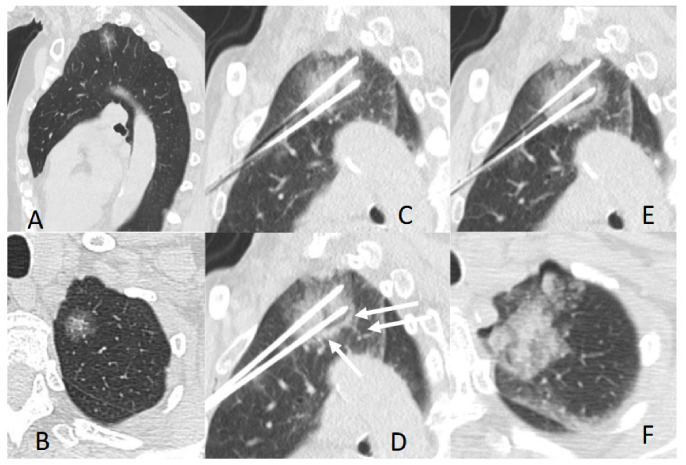
(**A**,**B**) Sagittal and axial CT scan images of biopsy-proven left apical adenocarcinoma with lepidic pattern with a maximum diameter of 28 mm in the craniocaudal dimension. (**C**) A sagittal reconstruction image with two cryoprobes placed at the edges of the tumor approximately 2 cm apart (1-2 rule). The current scan is at end of a 3 min freeze cycle. The ice ball is not seen during the freeze cycle and is best visualized during the thaw cycle. (**D**) An image following 3 min of thaw cycle. The edge of the melting ice ball is seen as a hemorrhage and a rim of consolidation (arrow). (**E**) Cryoablation at the completion of 3 min freeze, 3 min passive thaw, 5 min freeze, 3 min passive thaw, 7 min of freeze. (**F**) Following completion of cycles, active thaw was performed and the probes were removed. Post-ablation hemorrhage is surrounding the primary tumor.

All ablation procedures were performed by one of three experienced interventional chest radiologists (SG, RS, FA) with 5 to 20 years of experience in IGTA. Patients were positioned in a prone or supine position to ensure optimal access to the target lesions. Conscious sedation or general anesthesia was used depending on the case complexity or operator preference. Under standard sterile conditions, the ablation needle was advanced percutaneously using conventional CT guidance (Siemens Sensation 64 or Siemens Definition, Siemens Healthineers, Forchheim, Germany). For each ablation procedure, the probes or antennae were advanced incrementally to finally extend 5 mm beyond the deep margin of the tumor, and then ablation settings were adjusted based on the ablation maps provided by the manufacturer and targeted to incorporate at least a 5 mm margin beyond the edge of the target tumor. All MWAs were performed using PR-15 Antennae (NeuWave, Madison, WI, USA) and RFA was performed with a cooled tip single-electrode system (Coviden, Boulder, CO, USA). MWA and RFA were performed on tumors which were at least 1.5 cm away from the pleura, near larger vessels, and in central locations and in patients with coagulopathies and on blood thinners. For MWA and RFA, an immediate post-ablation scan was used to observe the halo and ground-glass surrounding the tumor to confirm adequate ablation coverage and technical success, as shown in Figure 2. Cryoablation was performed using two different systems—Endocare (Varian, Palo Alto, CA, USA) and Visual Ice (Boston Scientific, Marlborough, MA, USA)—and the respective cryoablation probes, namely Perc 24 and Perc 17 for the Endocare system and Ice Rod and Ice Edge for Visual Ice. Cryoablations were performed with multiple cryoablation cycles and adapted from modified triple-freeze cycles previously described in porcine models [18]. The choice of manufacturer used was dictated by the location of the procedure being performed and the availability of the device at the site. Intermittent intraprocedural CT scans at the end of cryoablation and thaw cycles were used to demonstrate the edge of the ice ball and plan the next cryoablation cycle, as well as to confirm extension of the ice ball by at least 5 mm beyond the edge of the tumor. When multiple probes were used, they were placed 1 cm from the edge of the tumor and 2 cm apart, referred to as the 1-2 rule (Figure 3). Cryoablation was preferred for peripheral tumors close to the pleura, fissures, or mediastinal pleura and for patients with implantable defibrillators or pacers [3,19,20]. A post-ablation CT scan was performed at the end of procedure and following removal of the antennae or probes to identify any potential treatment-related adverse events (AEs).

### 2.3. Follow-Up and Outcomes

Patients were monitored with a 1-week follow-up chest X-ray (CXR) and office visit and then followed with CT scans, preferably with contrast, and office visit at 1, 3, 6 and 12 months and thereafter at 6–12 months intervals for up to 60 months from the last ablation. Annual PET/CT scans were performed instead of the 12-month CT scan, to assess the metabolic activity of the ablated nodules for local recurrence and potential distant metastasis. The assessment of ablated lung tumors was based on the size, geometry, enhancement characteristics and FDG activity. Tumor response was evaluated using modified response criteria set forth in the SOLSTICE trial, using the 1-month scan as the baseline and tumor response was calculated by comparing the largest diameter of ablation zones. “Complete” response of index tumor(s) was defined as disappearance of the tumor ablation zone or a reduction of at least 75%; “partial” response as a 30% to 75% decrease in size; “stable disease” as less than 30% decrease and less than 20% increase in size; and “local failure” as an increase of greater than 20% compared with the smallest diameter (nadir) or appearance of nodular enhancement. Local tumor effectiveness includes complete, partial, and stable treatment response [21]. A thin rim of peripheral enhancement on CT scan or mild peripheral rim of FDG uptake on FDG PET scan within 6 months post-ablation were considered as post-ablation changes and not a viable tumor [17].

The safety assessment included evaluation of treatment-related AEs occurring within one month of the ablation. AEs were assessed based on the Society of Interventional Radiology classification system, and immediate AEs within 24 h, early AEs within 30 days and delayed AEs beyond 30 days were recorded [22]. Multiple endpoints to assess changes in clinical course as result of IGTA were used [23]. The primary outcomes were to measure 5-year OS, PFS, and CSS. The secondary outcomes were technical success and treatment-related AEs when thermal ablation was used for the treatment of single tumors or MPLC.

### 2.4. Statistical Analysis

Summary statistics were provided for demographics and lung cancer characteristics by single and multiple lung cancers. Wilcoxon rank-sum test for continuous variables and Fisher’s exact test for categorical variables were used to compare various patient demographics and lung cancer characteristics between single and multiple lung cancers. Kaplan–Meier analysis with the log rank test was used to estimate OS, PFS, and CSS survival curves. Time to event was calculated from the date of tissue diagnosis for single tumors and from the initial biopsy for MPLC. Multivariable Cox proportional hazards regression models were used to evaluate the association between multiple versus single lung cancers with 5-year progression-free survival (PFS), overall survival (OS), and cancer-specific survival (CSS). The multivariable CSS model is limited by sparse events (n = 3 cancer-specific deaths), resulting in wide confidence intervals and potentially unstable estimates; CSS findings from the adjusted model should be interpreted with caution. Models were adjusted for sex, age, tumor T-stage at study entry, history of radiation therapy, history of surgery, and history of chemotherapy. Hazard ratios (HRs) with 95% confidence intervals (CIs) were reported. The proportional hazards assumption was assessed using Schoenfeld residuals. *p* values of less than 0.05 were regarded as statistically significant. All statistical analyses were performed using Stata (version 18.0; StatCorp, College Station, TX, USA).

## 3. Results

A total of 37 patients (mean ± SD age = 71.6 ± 8.8) underwent a total of 119 IGTAs, with a single ablation procedure for 14 (37.8%) patients with single lung cancer and 105 ablations performed in 88 ablation procedures for 23 (62.2%) patients with MPLC. A maximum of three tumors were ablated per ablation procedure in two patients, and two tumors per ablation procedure in five patients, and the rest involved ablation of one tumor per ablation procedure. The maximum diameter of the tumors was mean (SD) of 16.3 mm (±7.40) with a range of 6–32 mm. The mean time between the first and last ablation procedures was 37 months (a range of 2–80 months). All ablations were completed successfully. The specific MWA and cryoablation techniques are displayed in Figure 2 and Figure 3, respectively. Patient characteristics demonstrated no significant differences between single lung cancer and MPLC in age (*p*= 0.5882), sex (0.732), race (0.763), ethnicity (0.765), history of chemotherapy (0.420), history of radiation (0.823) and history of surgery (0.582), as shown in Table 1. In addition to primary lung cancer, 12 patients had a history of malignancies other than lung cancer. The tumor characteristics are demonstrated in Table 2 with adenocarcinoma being most common tumor (89.2%), and even more common in MPLC (91.3%). The most common lobe for treated single lung cancer was the left lower lobe (28.6%) and for MPLC the right upper lobe (30.4%) and the most common T classification for both single lung cancer and MPLC was T1B at 50.1% and 44.7% respectively. The ablation parameters are described in Table 3 with the maximum diameter of tumors (mm) ± SD (range) measuring 16.3 ± 7.4 (6–32).

Mild AEs including self-limiting pneumothorax, transient dyspnea and pain were frequent and were managed with supportive care. Severe AEs included pneumothorax requiring hospital admission and intrapleural catheter insertion occurred in 5.8% (7/119) of the ablations. No other moderate or severe AEs were observed immediately after the ablation. Mortality rates as a result of ablation at 30 days and 90 days after the ablation procedure were 2.7% (1/37) and 5.4% (2/37), respectively. This included one patient whose cause of death was acute respiratory failure following ablation and another from end-stage lung disease and hemopneumothorax after ablation. Local recurrence was observed in 4/119 (3.3%) tumors ablated with three recurrences in the MPLC cohort and one in the single lung cancer cohort. All four recurrences were for central tumors following MWA with tumors ranging from 13 to 11 mm in long-axis diameter. Local recurrences were managed as follows: two were successfully treated with microwave ablation (MWA) and cryoablation (CA); one required two sessions of MWA to achieve remission; and the fourth was treated with a combination of MWA and subsequent radiotherapy. The causes of death varied and related mostly to comorbidities and concurrent cancers and are demonstrated in Table 4.

**Figure 4:**
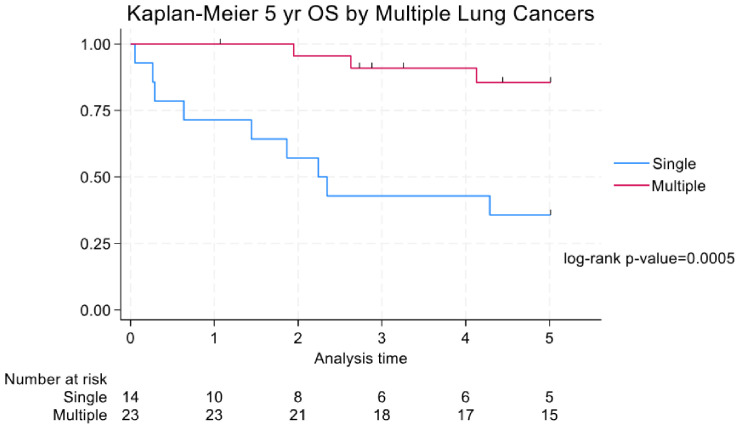
A Kaplan–Meier curve for 5-year OS by number of lung cancers (OS = overall survival).

**Figure 5:**
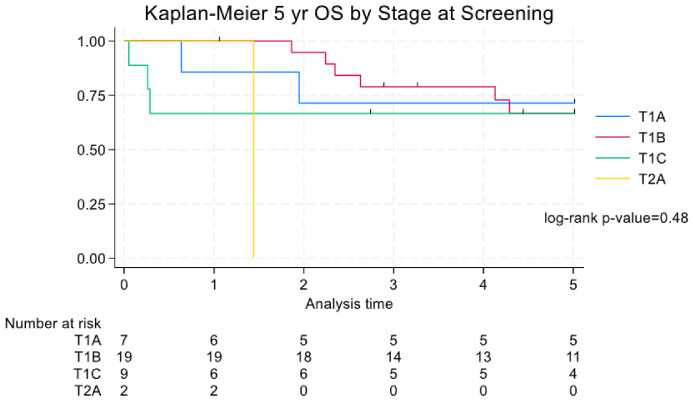
A Kaplan–Meier curve for 5-year OS by stage at screening (OS = overall survival).

The OS rates following IGTA for single lung cancer and MPLC were 71.4% (10/14) and 95.7% (22/23), respectively, at one year, 42.9% (6/14) and 77.4% (15/23), respectively, at three years, and 35.7% (5/14) and 85.6% (15/23), respectively, at five years with a hazard ratio (HR) (SE) of 0.14 (0.092) and confidence interval (CI) of 0.037–0.51 (Figure 4). When viewing 5-year OS within the context of the T stage of cancer, there was no significant difference for stages T1A, T1B, and T1C, at 71.4% (5/7), 66.8% (11/19) and 66.7% (4/9), respectively. However, patients with stage T2A had 0% (0/2) survival beyond two years, which may be related to the small sample size or the size of the tumor (Figure 5).

**Figure 6:**
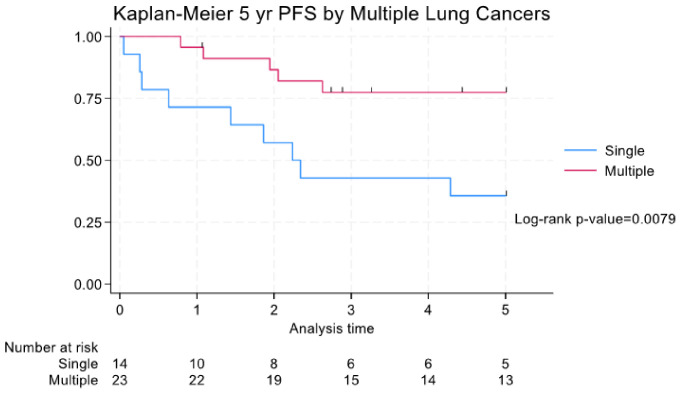
A Kaplan–Meier curve for 5-year PFS by number of lung cancers (PFS = progression free survival).

**Figure 7:**
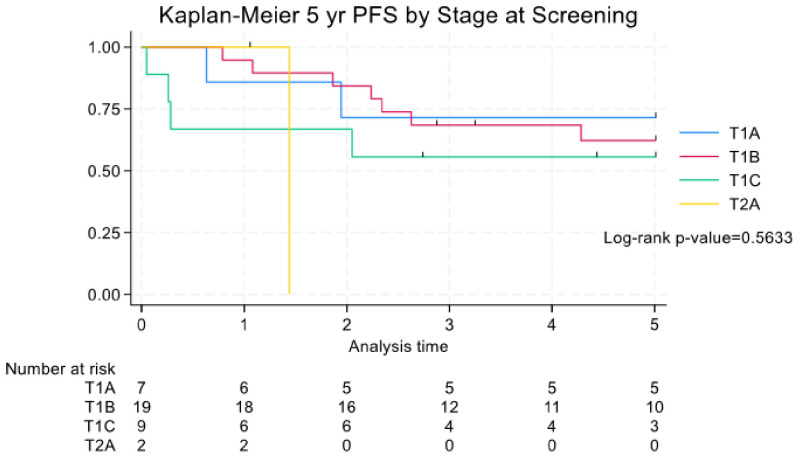
A Kaplan–Meier curve for 5-year PFS by stage at screening (PFS = progression free survival).

The PFS for single lung cancer and MPLC were 71.4% (10/14) and 95.7% (22/23), respectively, at one year, 42.9% (6/14) and 77.4% (15/23), respectively, at three years, and 35.7% (5/14) and 77.4% (13/23), respectively, at five years with a HR (SE) of 0.25 (0.14) and a CI of 0.084–0.76. (Figure 6). When viewing PFS within the context of the stage of cancer at diagnosis at stages T1A, T1B, and T1C, there was a gradual decrease in the five-year PFS rates, which were 71.4% (5/7), 62.2% (10/19) and 55.6% (3/9), respectively. The PFS for T2A patients was 0% at two years and beyond with a HR (SE) of 4.27 (5.40) and a CI (0.36–50.93) (Figure 7).

The CSS for single lung cancer and MPLC were 92.3% (10/14) and 100% (23/23), respectively, at one year, 83.1% (6/14) and 95.2% (18/23), respectively, at three years, and 83.1% (5/14) and 95.2% (15/23), respectively, at five years with a HR (SE) of 0.21 (0.26) and a CI of 0.018–2.33. The CSS at five years for stages T1A, T1B, and T1C, were 100% (5/7), 93.8% (11/19) and 87.5% (4/9), respectively. Patients with stage T2A had a CSS of 0% beyond the second year.

After adjusting for clinical variables, MPLC had significantly reduced risk of PFS by 91.4% (*p*-value = 0.001) and OS by 95.6% (*p*-value = 0.001) when compared to SLC but did not achieve statistical significance for CSS, Table 5.

## 4. Discussion

This study examined the long-term outcomes in a cohort of 37 patients who underwent IGTA(s),including CA (n = 63), MWA (n = 52) and RFA (n = 4) for single lung cancer and MPLC. The treatment of early-stage single lung cancer with IGTA has been shown to be effective but data is limited on MPLC. Multiple single-institution observational studies and a few prospective multi-institutional studies show a range of survival outcomes for single lung cancer. Moore et al. demonstrated a 5-year survival rate for stage 1A lung cancer at 67.8% ± 15.3 [24]. The RAPTURE (radiofrequency ablation of pulmonary tumors response evaluation) trial included 33 patients with NSCLC (13 with stage 1 disease) and reported a 2-year overall survival of 48% [25]. The Alliance trial (American College of Surgeons Oncology Group-sponsored Z4033) included 2-year outcomes from 51 patients from 16 institutions where RFA was utilized to treat medically inoperable patients with stage IA (T1a-bN0) NSCLC with a two-year overall survival of 70%, and a recurrence-free survival of 60% [4]. When compared to surgery, multiple retrospective studies of patients with stage 1 NSCLC have shown that survival rates following ablation do not differ from those who undergo sublobar resection [26,27]. Multiple retrospective propensity-matched registry studies have compared survival outcomes of IGTA to SABR and shown no significant difference in overall survival [28]. Uhlig et al. reviewed 28,834 patients with stage I lung cancer and compared overall survival between SABR and IGTA, reporting no significant difference between the two cohorts: 26% for SABR versus 25% for IGTA (*p* = 0.81) [29].

However, the role of IGTA in the treatment of MPLC has not received much attention. In recent years with increased use of imaging and propagation of lung cancer screening, more MPLC cases are being identified. MPLC cases are considered to arise separately from anatomically distinct malignant progenitor cells that independently undergo different genetic alterations, leading to neoplastic transformation [30]. Evaluation of the entire bronchial tree surrounding a primary bronchial squamous cell carcinoma showed allele-specific imbalances and widespread genetic changes in the whole lung [30]. Sikkink et al. proposed that multifocality represents clonal expansion of multiple separate primary neoplasms arising from a background of field cancerization [31]. Multifocal clonal lineage analysis in MPLC, and its differentiation from intrapulmonary metastasis, has improved with next-generation sequencing (NGS). Advances include large, targeted gene panels, whole-genome sequencing (WGS), and assessment of chromosomal rearrangements, along with evolving analytic approaches [32].

The International Association for the Study of Lung Cancer (IASLC) recognizes multiple sites of primary cancer including secondary primary lung carcinoma and multifocal lung adenocarcinoma with ground-glass or lepidic patterns. The demographic characteristics, outcomes, and recurrence patterns for secondary lung carcinomas are similar to those of single “typical” lung cancers according to stage and histologic type. Multiple lung cancer nodules with prominent ground-glass or lepidic (GG/L) features, have different demographic characteristics, excellent outcomes, and infrequent recurrences outside the lung parenchyma and hence are associated with better survival outcomes confounding the results [16].

IGTA carries some advantages for the treatment of MPLC over SABR and surgery. IGTA has been shown to preserve pulmonary function (PF), as shown on RAPTURE trial [25] and ACSOG Z403, preserving the PF parameters of forced expiratory volume at 1 s (FEV1), forced vital capacity (FVC) and diffusion capacity of the lung for carbon monoxide (DLCO) at 24 months, and other studies have shown no significant decline in short-term (1–3 months) or long-term (1–2 years) lung function with preservation of DLCO and spirometry at 24 months post-treatment, despite destruction of lung parenchyma; hence, IGTA can be performed multiple times as demonstrated in this cohort with 59.5% of patients undergoing multiple IGTA procedures [33,34]. IGTA also carries the benefit of shorter hospital stay and lower costs to the health system [35]. The use of thermal ablation for recurrent NSCLCs following surgical resection or radiotherapy has also been established with success by other investigators [5]. Here we used IGTA for treatment of recurrence after IGTA as well. Although there was a limited number of 4/119 (3.3%) recurrences of ablations, all recurrences occurred following MWA and in central tumors. As per technical protocol central tumors were recommended to undergo MWA to overcome the heat sink from large vessels. This confounding factor could have contributed to recurrence after MWA rather than the modality itself. The size of the recurrent tumors—ranging from 11–13 mm and all T1B—is less likely to have contributed to recurrence as there was no recurrence following T1c tumors.

In this cohort, patients with MPLC had better survival than those with single lung cancer on all survival metrics, namely OS, PFS and CSS (Figure 4, Figure 5, Figure 6 and Figure 7). Comparing the demographic variables between single lung cancer and MPLC, there was no significant difference in age, sex, race or ethnicity, as shown in Table 1. Also, there was no difference in prior therapies including history of chemotherapy, radiation or surgery, as shown in Table 1. Hence the difference in survival could be attributed to the nature of the MPLC as these tumors demonstrated a more indolent disease and may be subject to immortal time bias. Another issue that can affect survival is the frequency of surveillance among the single lung cancer and MPLC groups, although in this study it is not clear which group may have received more rigorous surveillance CT scans. The better survival outcomes in this study could also be from a predominant adenocarcinoma cohort (94.5%) which is an independent predictor of good outcome [36,37]. Multiple previous studies have shown the 5-year OS for treated MPLC to be ranging from 54.8% to 81.1% which confirms heterogeneity of this cohort and the need for a better standardized description [36,37,38]. However, contrary to the present study, a few meta-analyses of resected NSCLC patients with and without synchronous primary lung carcinoma found no significant difference in survival [38]. 

Following IGTA, the size of the tumor up to 3 cm (T1c) did not affect the OS with a trend towards better survival for smaller tumors for T1a, T1b and T1c at 71.4%, 62.2% and 55.6% respectively, but once the tumors grew larger than 3 cm (T2A) there was a significant drop in OS of 0% at 2 years and beyond, although the number of patients (n = 2) limited a more in-depth evaluation, as shown in Figure 5.

In this study, the 5-year CSS for single lung cancer and MPLC was 83.1% and 95.2% respectively, with a total of 12/37 deaths after a median follow-up of 44 months, of which three were directly related to lung cancer. The present study’s results fare better for the treatment of single lung cancer when compared to the literature. A study of 80 RFA procedures in 59 patients with stage 1 NSCLC who were medically inoperable reported CSS of 89% at 1 year, 59% at 3 years, and 40% at 5 years post-ablation [39]. Moore et al. found the 5-year cancer-specific survival of all patients treated for single lung cancer, where cancer recurrences and all deaths were counted as events, to be 56.6% ± 16.5 [24].

The present study’s main AE was pneumothorax. Major pneumothorax occurred following seven procedures (5.8%). These patients had a prolonged hospital stay, and one patient died shortly after ablation. One other patient died from post-ablation respiratory failure more than one month after thermal ablation. This cohort included patients with multiple comorbidities, and mortality in the cohort was attributed to conditions unrelated to the primary lung malignancy, such as advanced dementia, exacerbation of COPD, and pneumonia. Twelve individuals had additional sources of primary malignancies other than lung cancer, including lymphoma, breast, pancreas and prostate cancers among others, theoretically impacting the OS (Table 4).

## 5. Limitations

This study was subject to some limitations. First, it mostly considered primary lung tumors mostly with a mean diameter of ≤3 cm. A significantly decreased rate of short- and long-term survival has been reported in tumors > 3 cm compared to smaller tumors following thermal ablation of lung tumors [8,11]. It has been suggested that MWA might be utilized to treat lung tumors with initially larger diameters. Following MWA of single primary or metastatic lung cancers, Healey et al. reported an odds ratio of 11.1 (95% confidence interval: 2.97–41.1) for primary technical success in tumors < 3 cm compared to larger tumors [8]. Second, this is a single-center retrospective study, and the results are influenced by the operators’ level of experience. Therefore, the findings may not be fully generalizable. In addition, the use of RFA was limited compared with MWA and cryoablation, largely reflecting a preference shift from RFA to MWA over time. Third, not all MPLC exhibit uniform biological behavior or identical survival outcomes, even when untreated, which may impact overall survival results. The biopsy results were limited to histology results and molecular biomarkers were not included in the analysis, which in turn could have contributed to the survival results. The fourth limitation is the grouping of nodule sizes within the single lung cancer and MPLC cohorts which introduces confounding when addressing survival metrics. Patients with MPLC may have had a more indolent type of cancer than patients with single lung cancer. The possibility of indolent MPLC is an important confounder for survival and introduces immortal time bias. Also, for MPLC, not all the nodules were biopsied to confirm presence of NSCLC and to distinguish another primary from metastatic disease; however, rigorous review was performed by a multidisciplinary tumor board, as described in the Section 2, to minimize this limitation and capture nodules which best represented lung carcinoma. Finally, the number of lung cancers treated was relatively small, as most early-stage cases are managed with surgical resection or SABR, making it challenging to access patients at initial presentation.

## 6. Conclusions

IGTA of primary lung carcinomas is a safe and effective treatment in patients with single and multiple stage 1 non-small cell lung carcinoma, with limited local recurrence. The OS, PFS and CSS rates in patients with MPLC are better than those in patients with single lung cancer, but are only statistically significant for OS. Tumor size within the stage 1A group did not affect the survival metrics.

## Figures and Tables

**Table 1:** Demographics of the patient cohort.

Characteristic	Total (N = 37)	Single Primary Lung CA (N = 14)	Multiple Primary Lung CA (N = 23)	*p*-Value
Age, mean (SD)	71.6 (8.8)	73.3 (9.3)	70.5 (8.5)	0.5882
Sex, N (%)				0.732
Female	23 (62.2)	8 (57.1)	15 (65.2)	
Male	14 (37.8)	6 (42.9)	8 (34.8)	
Race, N (%)				0.763
Asian	4 (10.8)	1 (7.1)	3 (13.0)	
White	31 (83.8)	13 (92.9)	18 (78.2)	
Other	1 (2.7)	0	1 (4.4)	
Unknown	1 (2.7)	0	1 (4.4)	
Ethnicity, N (%)				0.765
Hispanic	4 (10.8)	1 (7.1)	3 (13.0)	
Non-Hispanic	32 (86.5)	13 (92.9)	19 (82.6)	
Unknown	1 (2.7)	0	1 (4.4)	
History of chemo, N (%)				0.420
No	19 (51.4)	6 (42.9)	13 (56.5)	
Yes	18 (48.6)	8 (57.1)	10 (43.5)	
History of radiation, N (%)				0.823
No	22 (59.5)	8 (57.1)	14 (60.9)	
Yes	15 (40.5)	6 (42.9)	9 (39.1)	
History of surgery, N (%)				0.582
No	18 (48.7)	6 (42.9)	12 (52.2)	
Yes	19 (51.3)	8 (57.1)	11 (47.8)	
Cause of death, N (%)				1.000
Cancer	3 (8.1)	2 (14.3)	1 (4.4)	
Other	9 (24.3)	7 (50.0)	2 (8.7)	
No death	25 (67.6)	5 (35.7)	20 (86.9)	

**Table 2:** Lung cancer characteristics, staging, and treatment type.

Lung Cancers, N (%)	Total (N = 37)	Single (N = 14)	Multiple (N = 23)	*p*-Value
NSCLC Type, N (%)				0.625
Adenocarcinoma	33 (89.2)	12 (85.7)	21 (91.3)	
Squamous cell CA	4 (10.8)	2 (14.3)	2 (8.7)	
Nodule location, N (%)				N/A
	(n = 119)	(n = 14)	(n = 105)	
Left upper lobe:	23 (19.3)	3 (21.4)	20 (19.1)	
Left lower lobe:	25 (21.0)	4 (28.6)	21 (20.0)	
Right upper lobe:	35 (29.4)	3 (21.4)	32 (30.4)	
Right middle lobe:	4 (3.4)	1 (7.1)	3 (2.9)	
Right lower lobe:	32 (26.9)	3 (21.4)	29 (27.6)	
TNM staging by initial target tumor, N (%)				1.000
T1A	7 (18.9)	3 (21.4)	4 (17.4)	
T1B	19 (51.4)	7 (50.1)	12 (52.1)	
T1C	9 (24.3)	3 (21.4)	6 (26.1)	
T2A	2 (5.4)	1 (7.1)	1 (4.4)	
TNM staging by each target tumor, N (%)	(n = 119)	(n = 14)	(n = 105)	N/A
T1A	46 (38.7)	3 (21.4)	43 (41.0)	
T1B	54 (45.4)	7 (50.1)	47 (44.7)	
T1C	16 (13.5)	3 (21.4)	13 (12.4)	
T2A	3 (2.5)	1 (7.1)	2 (1.9)	
Ablation type per nodule, N (%)	(n = 119)	(n = 14)	(n = 105)	N/A
Cryoablation	63 (52.9)	4 (28.6)	59 (56.2)	
MWA	52 (43.6)	7 (50.0)	45 (42.9)	
RFA	4 (3.4)	3 (21.4)	1 (1.0)	

**Table 3:** Ablation parameters used for treatment of single lung cancer and MPLC.

Size of Tumor	
Maximum diameter mean ± SD (range)	16.3 ± 7.4 (6–32)
Mean diameter (range)	13.4 + 6.6 (8–29)
**Cryoablation: total**	63/119 (53%)
Number of freeze cycles: 3, N (%)	61 (97%)
Number of freeze cycles: 4, N (%)	2 (3%)
Total freeze cycle time (in minutes)	17–30
Maximum time of longest freeze cycle time (in minutes)	12
Thaw between cycles time (in minutes)	1–3
**Microwave ablation**	52/119 (44%)
65 W, N (%)	37 (75%)
45 W, N (%)	11 (21%)
30 W, N (%)	2 (3%)
Mean time (range) (in minutes)	8 (5–10)
1 antenna per ablation	34 (65%)
2 antennas per ablation	18 (35%)
**Radiofrequency ablation**	4/119 (3%)
70 W, N (%)	4 (100%)
Two 10-minute cycles, N (%)	4 (100%)

**Table 4:** Cause of death in the deceased patient subgroup.

Patient Number	Cause of Death	Month After Initial Ablation
1	Post-ablation respiratory failure	1
1	Hospice care for pancreatic cancer	3
1	Acute respiratory failure from end-stage lung disease and hemopneumothorax after ablation	3
1	Died on hospice of advanced lymphoma	9
1	Died on chemotherapy for metastatic lung cancer	17
1	Advanced SLE, passed away in sleep	24
1	Advanced ALS	26
1	Acute pneumonia	28
1	Acute respiratory failure after stroke	32
1	COPD with acute exacerbation	50
1	Advanced dementia	52
1	Unknown cause of death	77

**Table 5:** The table demonstrates multivariable Cox proportional hazard models for PFS, OS and CSS.

Characteristics n = 37	5-year PFS	5-year OS	5-year CSS
	HR (SE)	HR (SE)	HR (SE)
	[CI]	[CI]	[CI]
Multiple Primary Lung Cancer vs. Single Lung Cancer	0.086 (0.066)	0.044 (0.041)	0.22 (0.28)
	[0.019–0.39]	[0.0071–0.27]	[0.019–2.66]
	*p*-value = 0.001	*p*-value = 0.001	*p*-value = 0.24

## Data Availability

The original contributions presented in this study are included in the article. Further inquiries can be directed to the corresponding author.
